# Protocol for a systematic review of the factors associated with binge drinking among adolescents and young adults

**DOI:** 10.1186/s13643-017-0461-3

**Published:** 2017-04-11

**Authors:** Jennifer Hasselgård-Rowe, Barbara Broers, Dagmar M. Haller

**Affiliations:** 1 Primary Care Unit, Faculty of Medicine, University of Geneva, UIGP, 1 rue Michel-Servet, 1211 Geneva 4, Switzerland; 2 Division of Primary Care, Department of Community Medicine, Primary Care and Emergency, Geneva University Hospitals, 6 rue Gabrielle-Perret-Gentil, 1211 Geneva-14, Switzerland; 3 Primary Care Physician, Youth Health Clinic, UIGP, 1 Rue Michel-Servet, 1211 Geneva 4, Switzerland

**Keywords:** Adolescent, Young person, Binge drinking, Risky single occasion drinking, Heavy episodic drinking, Risk factors

## Abstract

**Background:**

Alcohol use is the behaviour that has the most significant impact on the mortality and morbidity of young people, and binge drinking is becoming the norm among this population. The burden of disease of binge drinking during adolescence and young adulthood is significant and warrants the development of effective prevention strategies. Although the literature on risk factors for excessive substance use has been increasing, to our knowledge, a systematic review of the factors associated with binge drinking among young people has not yet been conducted. This study aims to identify and organise the factors associated with binge drinking among young people aged 15 to 24 years; and to provide a framework to further understand these factors in order to better target prevention strategies.

**Methods/design:**

This systematic review of the literature will follow the Preferred Reporting Items for Systematic Reviews and Meta-Analyses (PRISMA) recommendations. The databases PubMed, Embase, PsycINFO and Social Care will be searched for articles published between 1 January 2006 and 31 December 2015. Our search focuses on studies examining the risk factors for binge drinking among young people (between the ages of 15 and 24). Observational studies (cross-sectional, cohort and case-control studies) will be included, while randomised controlled trials will be excluded. Case series and case reports will also be excluded, while reviews, if relevant, will be included. The primary outcome is binge drinking. Secondary outcomes include indicators of frequency and consequences of binge drinking. Two reviewers will independently screen articles, extract relevant data and assess the quality of the studies.

**Discussion:**

This systematic review will add to our knowledge and understanding of binge drinking among young people. It will allow us to identify the main risk and protective factors associated with binge drinking among this population and ultimately help to define the lines for further investigation and research, as an important part of prevention strategies in this area of work.

**Systematic review registration:**

This protocol is registered in the PROSPERO registry of the University of York (reference number: CRD42016032496).

**Electronic supplementary material:**

The online version of this article (doi:10.1186/s13643-017-0461-3) contains supplementary material, which is available to authorized users.

## Background

Alcohol use is the behaviour that has the most significant impact on the mortality and morbidity of young people [1], and binge drinking is becoming the norm among this population [2]. Excessive cannabis and/or alcohol use in adolescence has many negative short- and long-term consequences which have already been well documented [3, 4]. In high-income countries like Switzerland, the burden of disease, measured by DALY’s (disability adjusted life years) increases significantly from early to late adolescence, particularly with regard to mental health and substance use issues [1].

Although the definition for ‘binge drinking’ varies, a common one found throughout the literature refers to a binge drinking episode as the consumption of five drinks (four for females) or more on one occasion [5–8]. Binge drinking, on at least a monthly basis, in adolescence and young adulthood is associated with a higher risk of alcohol dependence and psychosocial difficulties later in life [5, 9, 10]. Worryingly, young people who engage in binge drinking also have a risk 5 to 10 times greater than others of spending time in hospital emergency rooms as a result of their excessive alcohol use [11]. For these reasons, prevention strategies aimed at pushing back the age at which adolescents have their first taste of alcohol as well as reducing the levels of alcohol intake among adolescents who already drink have become a priority (Haller et al. 2014) [2].

With regard to risk factors influencing binge drinking among adolescents, Kuntsche et al. (2004) [4] conducted a review of the socio-demographic, individual and social factors that affect binge drinking in 2004. Although the study provides a good starting point for this review, the publication is now 12 years old, and given the changes in the extent of binge drinking, particularly its normalisation in many Western societies, it remains highly relevant to revisit the key risk and protective factors associated with binge drinking among adolescents and young adults. Moreover, despite the fact that the literature on risk factors for excessive substance use has been increasing [12, 13] to our knowledge a systematic review of the factors associated with binge drinking among young people has not yet been conducted. This study aims to fill this gap and offer a framework for a better understanding of these factors so as to identify and inform effective prevention strategies.

### Aim

The overall aim of this systematic review of the literature is to provide a comprehensive overview of the various risk and protective factors for binge drinking among young people aged 15 to 24 years. The choice of this age range is based on the fact that as Sawyer et al. (2012) [14] note: ‘young people in this age group share a similar burden of disease throughout this development phase from adolescence into adulthood’, and with particular regard to binge drinking, the period between the ages of 15 and 24 is one of particular vulnerability in terms of short- and long-term negative consequences of this behaviour [5, 9]. Moreover, 15 is the age as from which substance use starts becoming really significant [15].

This study will identify particular adolescent subgroups that are more likely to engage in binge drinking. The aim is also to offer a framework to further understand these factors in order to better target prevention strategies. Indeed, greater attention to adolescents is crucial to the success of many public health goals [14]. This systematic review uses the life-course perspective expounded in Sawyer et al. (2012) [14] which emphasises that

the health of adolescents is affected by early childhood development and the biological and social-role changes that accompany puberty, shaped by social determinants of health that affect the uptake of health-related behaviours. The onset of these behaviours and states in adolescence affect the burden of disease in adults and the health and development of their children.

## Methods/design

### Objectives

The primary objective is to identify and summarize the factors associated with binge drinking among adolescents and young adults (15 to 24 years old).

### Search strategy

A systematic review of the literature will be conducted. The following bibliographic databases will be searched for articles published in the last 10 years, (between 1 January 2006 and 31 December 2015):

- PubMed
- Embase
- PsycINFO
- Social Care

The literature search strategy is included in Additional file 1. Our search focuses on studies examining the risk factors for binge drinking among young people (between the ages of 15 and 24 years). Two reviewers will independently screen articles, extract relevant data and assess the quality of the study by using the table of quality criteria of studies established by Glasziou et al. (2001) [16] and the Newcastle-Ottawa Scale) [17]. This protocol conforms to the Preferred Reporting Items for Systematic Reviews and Meta-Analyses Protocols (PRISMA-P) guidelines [18, 19], found in Additional file 2: Table S1. This protocol has been registered in the PROSPERO registry of the University of York.

### Eligibility criteria

PICOS (population, intervention (changed to exposure for the purposes of this review of observational studies), comparator, outcome, study characteristics) was used to define the eligibility criteria for this study.

#### Population

Studies will be considered if they contain information on binge drinking and its related factors and focus on young people between the ages of 15 and 24. For the purposes of this review, we use the cut-off of 25 years of age, in line with the United Nation’s definition of ‘youth’ which for epidemiological purposes defines “youth” as those persons between the ages of 15 and 24 years, ‘without prejudice to other definitions by Member States’ (United Nations (UN), *Definition of Youth*). The authors further specify that both “young people” and the composite term « adolescents and young adults » [20] are used to refer to people aged 10 to 24 years with data increasingly being divided into three categories: 10 to 14 years (early adolescence), 15 to 19 (late adolescence), and 20 to 24 years (early adulthood), so as to more accurately study the extent of health changes taking place during these phases [1, 5, 21]. According to these definitions, our study therefore concentrates on the two following sub-groups: late adolescents and young adults. Moreover, since young adults are sometimes considered in a continuum encompassing adult populations, studies involving participants older than 25 years old will also be included as long as the data related to our target age-group (15 to 24 years) can be analysed separately.

#### Exposure

The ‘exposure’ in this study refers to ‘factors’ which can be identified as associated with binge drinking. Therefore, studies that focus on the various risk or protective factors associated with binge drinking will be analysed. It is important for these factors to be anterior to the behavioural outcome of interest to this review: binge drinking. Most studies will no doubt be cross-sectional, measuring the risk and outcome at the same time. However, in terms of evaluating the causal relationship, the evidence will be stronger when the risk or protective factor is measured in longitudinal studies, rather than cross-sectional ones.

#### Comparator or control

By definition of the specific behaviour being studied, young people engaging in binge drinking will be compared to “unexposed young people”, in other words, young people who do not binge drink.

#### Outcomes

The primary outcome of interest to this review is binge drinking, also reported as ‘risky single occasion drinking’ or ‘heavy episodic alcohol use’. As noted above, there are various definitions of binge drinking, and for the purposes of this study, we will accept all definitions proposed in the articles we find. Since self-report of substance use (including binge drinking) has been shown to be a reliable measure in young people [22], studies measuring outcomes using any data source, including self-report will be eligible for inclusion.

Secondary outcomes for this systematic review will include the severity of binge drinking, through indicators of frequency and consequences of this behaviour in order to be able to stratify the risks in terms of their severity as part of the findings. Studies reporting on these primary and secondary outcomes and fulfilling the rest of the study design criteria will be included.

### Study characteristics

Observational studies (cross-sectional, cohort and case-control studies) will be included while randomised controlled trials (RCTs) will be excluded. Case series and case reports will also be excluded as they do not constitute sufficiently strong evidence. Where relevant, reviews will be included. Information found in books or letters will not be included. The articles may be in any language (but with an abstract in English), and the timeframe of the study covers articles published between 1 January 2006 and 31 December 2015.

### Search terms

The primary search question can be divided into three concepts, as follows:

- Concept 1: binge drinking (referring to the primary outcome of interest to this review);
- Concept 2: risk factors (referring to the ‘exposure’);
- Concept 3: adolescents (referring to the population the review is focusing on).

Additional file 3 illustrates these concepts, and the respective search terms used in the various databases.

### Definitions

The literature search strategy is included in Additional file 1. We shall use Medical Subject Headings (MeSH) and free-text word terms, as appropriate to the databases. With regard to the first concept, ‘binge drinking’ is classified as a PubMed MeSH term, and so it is used in the search equation in combination with free-text words such as ‘heavy episodic drinking’ and ‘risky single occasion drinking’.

For concept number 2, the MeSH terms ‘risk factor’ and ‘life style’ will be included, as will the free-text words ‘lifestyle’ (and life style). The term “risk behaviour” is not included as clustering of risk behaviours (sky-diving, drink driving, etc.) arising post excessive alcohol consumption is not the focus of this review, which instead is interested in identifying the factors which affect adolescents’ levels of alcohol consumption. Terms such as ‘depression’ , ‘anxiety’ , ‘psychological stress’ , ‘social isolation’ , ‘social desirability’ , although potentially constituting risk factors will not be included as individual search terms for two reasons: to avoid bias towards specific types of risks and on the assumption that studies examining these elements would, in any case, already be classified under ‘risk factor’ or ‘lifestyle’.

With regard to Concept 3, this study is specifically focused on binge drinking among young people, since previous research has established that excessive alcohol use within this population represents a heightened risk for alcohol dependence and psychosocial difficulties in adulthood [10]. In order to obtain as relevant results as possible, specific MeSH and most relevant free-text words will be used for concept 3 instead of the age-related filters that exist in some databases such as PubMed for example. The MeSH terms ‘young adult’ and ‘adolescent’ as well as free-text words such as ‘young adult’ , ‘young people’ , ‘young person’ , ‘underage’ , ‘juvenile’ , ‘youth’ and ‘teen*’ form part of the search equation. See Additional file 1 “Literature search strategy” for full details.

### Selection procedure

Selection of relevant studies will follow a three-step process, all carried out by two reviewers (JHR and DH). First, JHR and DH will independently screen the titles and abstracts of references collected for inclusion/exclusion criteria. Second, the full-text of the articles meeting the inclusion criteria on the basis of their abstract will be examined to make sure the articles should be included in the review. Third, the results will be compared, and any disagreements between the two reviewers will be resolved by discussion and consensus and, if need be, by submitting the questions to a third independent reviewer.

### Article selection

A data extraction form will be used to record the study authors, title of study and whether it should be excluded under one of the four exclusion criteria. These criteria are as follows:

1. Not binge drinking (or binge drinking less than once a month) even if the studies focus on alcohol consumption;
2. Not the type of study we are interested in (i.e. RCT or letters or books or case reports or case series);
3. The study does not focus on adolescents;
4. The ‘risk factors’ do not constitute risk or protective factors for binge drinking specifically even if they may be risk factors for other behaviours or activities. The timing of the risk factors of relevance to this study means they need to precede the binge drinking.

At each step of the selection process, reasons for inclusion/exclusion will be recorded in the PRISMA Flowchart [23], found in Additional file 4.

### Data extraction

Two authors (JHR and DH) will extract the data separately, in accordance with a data extraction sheet they have developed and pilot-tested on three articles. The elements to be extracted from each article include the kind of study, sample and intervention characteristics and the definition of binge drinking. This will also allow us to identify how binge drinking is defined and recorded in the various articles. Of particular note with regard to the timing: in the data extraction phase, we will take particular care with regard to information indicating that binge drinking is current and not past and that the measured risk factors were present before the levels of current binge drinking measured. Some cross-sectional studies may have measured previously occurring risk factors and newly occurring binge drinking, in which case we will obtain stronger evidence, while other cross-sectional studies will not provide information we can use, and it will only be possible to present the results from those kinds of studies in the form of associations.

### Data management

Bibliographic software (Endnote) will be used for the data management of retrieved references. All the results of the literature searches will be imported into the program and duplicates removed by the main reviewer (JHR).

### Data analysis and quality assessment

Once the final list of references has been established, the articles will be analysed. The quality of the observational studies identified in this review will be evaluated using a combination of two evaluation tools: the Newcastle-Ottawa Scale [17] (for case-control and cohort studies) as well as a quality scale adapted from Glasziou et al. (2001) [16] for cross-sectional studies. Criteria such as whether the study design was appropriate, how many participants were involved, the number and age of subjects, what are the main findings and what are the limitations of the study will be examined.

### Evidence synthesis

We will produce a narrative synthesis of the main results extracted from the full-text articles. A summary of included studies will provide information on the authors, study design, participants, number and age of subjects, theoretical framework (if relevant), alcohol consumption (as the primary outcome of interest), main findings as well as limitations or other interesting information about the study. Special emphasis will be placed on identifying and classifying the different risk or protective factors for binge drinking among young people. In the presentation of findings, we will try to separate factors for which the evidence of causality is strong (from longitudinal studies) and factors for which the causal nature of the relationship is less certain (cross-sectional data). A graphical summary of all the data representing and taking into account the number of studies providing evidence for a factor, and the relative strength of the presented association based on the study design and quality assessment will be provided. The level of association will be assessed based on adjusted data.

## Discussion

Globally, the increasing burden of disease due to non-communicable diseases has highlighted the need to pay greater attention to the health of adolescents [14]. Determining the risk (or protective) factors for binge drinking among adolescents and young adults is important in this regard. By identifying and classifying the factors associated with binge drinking among adolescents and young adults and offering a framework to better understand them, this systematic review will help identify effective prevention strategies which can help guide future avenues of research in this area. This study will help distinguish adolescent subgroups that are more likely to engage in binge drinking and contribute to creating better prevention programs tailored to various young persons’ needs. The results may, for example, be used to guide indicated screening in primary care. In addition to being important with regard to individuals’ health, the results of this study have the potential to inform and impact public health policies and development. This review will therefore contribute to better targeting of appropriate public health interventions as well as informing individual prevention tools. Given the lasting consequences that behaviours such as binge drinking among adolescents and young adults can have on individuals throughout their life, the impact of these strategies is potentially significant.

## Additional files

Additional file 1: “Literature search strategy” provides a description of the literature search strategy. (DOCX 14 kb)

Additional file 2: PRISMA P Checklist. (PDF 121 kb)

Additional file 3: Table S1. Concepts of the review and the list of search terms used in the literature search. (DOCX 14 kb)

Additional file 4: PRISMA Flowchart. (PDF 100 kb)

## Abbreviations

GPs: General practitioners
MeSH: Medical Subject Headings
PRISMA: Preferred Reporting Items for Systematic Reviews and Meta-Analyses
RCTs: Randomised controlled trials
