# Supplementary material for: Protocol for a systematic review of the factors associated with binge drinking among adolescents and young adults
Source: Syst Rev. 2017 Apr 11;6:76. doi: 10.1186/s13643-017-0461-3 (PMC5387323; doi:10.1186/s13643-017-0461-3)
Supplement: Supplementary file 1 — “Literature search strategy” provides a description of the literature search strategy. (DOCX 14 kb) [file 13643_2017_461_MOESM1_ESM.docx]

**Additional File 1**

**Literature Search Strategy for systematic review of the factors associated with binge drinking among adolescents and young adults**

**Database 1 (PubMed) search strategy:**

Search: ((((((((binge drinking/prevention and control[MeSH Terms]))) OR binge drinking/psychology[MeSH Terms]) OR heavy n2 drinking[Title/Abstract]) OR risky single occasion drinking[Title/Abstract])) AND ((((risk factors[MeSH Terms]) OR life style[MeSH Terms]) OR lifestyle[Title/Abstract]) OR life style[Title/Abstract])) AND (((((((young adult[MeSH Terms]) OR young adult[Title/Abstract]) OR young people[Title/Abstract]) OR ((adolescent[MeSH Terms]) OR adolescen*[Title/Abstract])) OR young n3 person*[Title/Abstract]) OR ((((teen*[Title/Abstract]) OR 'under age'[Title/Abstract]) OR 'underage'[Title/Abstract]) OR 'juvenile'[Title/Abstract])) OR ((youth*[Title/Abstract]) OR 'youth'[Title/Abstract]))

Filters: From 2006/01/01 to 2015/12/31, Humans
