# Supplementary material for: Protocol for a systematic review of the factors associated with binge drinking among adolescents and young adults
Source: Syst Rev. 2017 Apr 11;6:76. doi: 10.1186/s13643-017-0461-3 (PMC5387323; doi:10.1186/s13643-017-0461-3)
Supplement: Supplementary file 2 — PRISMA P Checklist. (PDF 121 kb) [file 13643_2017_461_MOESM2_ESM.docx]

**Table S1 : Concepts of the review and the list of search terms used in the literature search.**

| **PI(E)COS^7^** | **Keywords** |
| --- | --- |
| **Population**  **(concept 3)** | Adolescent, young adult, young people,, teen, teenager, juvenile, youth, underage. |
| **Exposure (concept 2)** | Risk factors, lifestyle (life style) |
| **Comparator / control** | Unexposed young people |
| **Outcome (concept 1)** | ‘Binge drinking’ or ‘risky single occasion drinking’ or ‘heavy episodic drinking’ |
| **Study design** | Observational studies (cross-sectional, cohort and case-control studies). |
