# Supplementary material for: Protocol for a systematic review of the factors associated with binge drinking among adolescents and young adults
Source: Syst Rev. 2017 Apr 11;6:76. doi: 10.1186/s13643-017-0461-3 (PMC5387323; doi:10.1186/s13643-017-0461-3)
Supplement: Supplementary file 4 — PRISMA Flowchart. (PDF 100 kb) [file 13643_2017_461_MOESM4_ESM.pdf]

PRISMA 2009 Flow Diagram

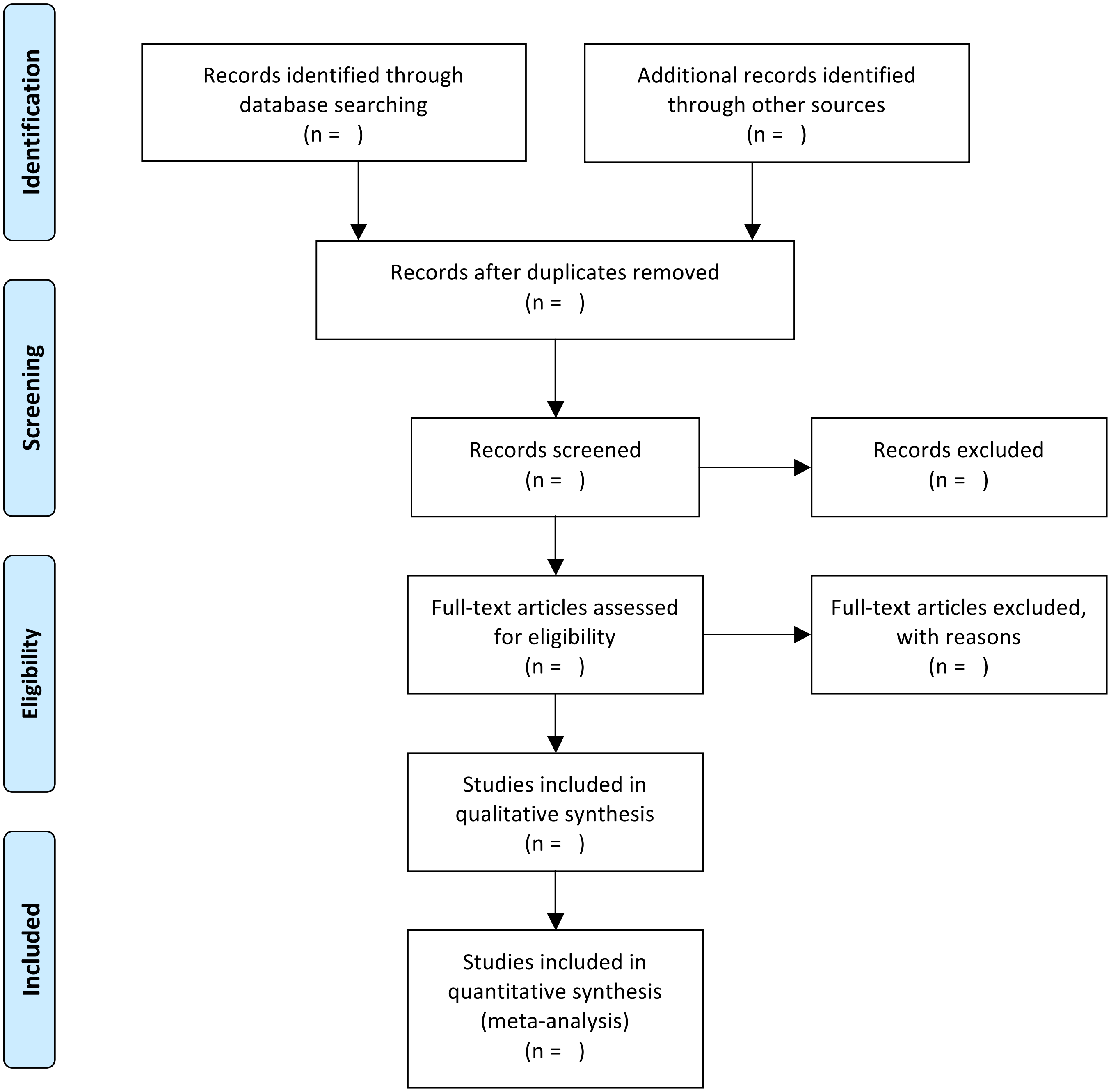

From: Moher D, Liberati A, Tetzlaff J, Altman DG, The PRISMA Group (2009). *P*referred *R*eporting *I*tems for *S*ystematic Reviews and *M*eta-*A*nalyses: The PRISMA Statement. PLoS Med 6(7): e1000097. doi:10.1371/journal.pmed1000097

For more information, visit www.prisma-statement.org.
